# Epidemic Dysentery

**Published:** 1858-03

**Authors:** William J. Johnson


ART. III.—*Epidemic Dysentery as it Occurred at Fort Gaines, Clay County, Georgia, during the Summer of* 1857; *with an Account of the Treatment Successfully Employed.* By WILLIAM J. JOHNSON, M.D.

DYSENTERY is a disease which can be traced back to the remotest period of antiquity. The writings of ancient medical authors contain accurate delineations of its symptoms, and elaborate histories and descriptions of its devastating ravages. In Sydenham's great work on *Acute and Chronic Diseases*, with notes by Dr. Rush, may be found an account of epidemic dysentery as it appeared in the city of London during the years 1669, 1670, 1671, and 1672. All the old writers on medical subjects regarded dysentery as a highly contagious disease, and hence, in their nosological arrangement, it was assigned a place among their "contagious pyrexia." The epidemic dysentery whose fearful ravages have been so severely realized, and whose "terrible rod" has scourged the people in portions of Southwestern Georgia and the adjacent counties of our neighboring sister State, Alabama, so painfully, and to a history of which I purpose occupying a few pages, was evidently, to a certain extent, contagious. "Facts are stubborn things;" and of this fact there cannot be entertained a reasonable doubt. The materies morbi generated by, and emanating from the bodies of sick patients, confined or closely crowded together in badly and imperfectly ventilated apartments, was capable—as the writer has had abundant means of verifying—of communicating the disease from one person to another until nearly entire families would become the subjects of its attack. So virulent was this poisonous agent in one of the families that I attended, and so malignantly fatal were the attacks, that I verily believe that nearly every member of the family would have fallen victims to the pestilence had this catastrophe not been averted by a timely separation of the diseased from the healthy.

*Symptoms.*—The attacks were usually ushered in by chills, or chilly sensations, or nervous rigor, with a general feeling of lassitude and *malaise.* This stage was followed by fever; the pulse was irritable, quick, and depressed; the thirst was intense; the heat about the head and over the abdomen was inordinate, with a coolness of the extremities. As soon as reaction came on—in many cases while it was being established—the patient would experience griping sensations in the bowels, accompanied by a bearing-down feeling, and a frequent inclination to void the fœces, which latter were chiefly mucus, sometimes mixed with blood, the natural contents of the intestines being retained, or passed in small compact scybalous masses. With these symptoms there was complete anorexia, and very frequently nausea and vomiting; the stomach immediately rejecting all substances, medicinal or otherwise, and thus the sufferings of the patient were greatly augmented, and the treatment correspondingly embarrassing to the physician. As the inflammation increased in intensity, the stools became more frequent and scanty, and in their passage over the phlogosed mucous membrane they occasioned a most intolerable pain, every evacuation being preceded or accompanied by severe griping, and sometimes by a rumbling or "growling" noise. The motions varied both in color and consistence, being sometimes composed of frothy mucus, mixed with blood; sometimes they were sanguinolent, pure blood being voided, and sometimes resembled the washings of fresh meat, and possessed a peculiarly offensive odor; sometimes muco-sanguinolent and totally inodorous. Occasionally lumps of coagulated mucus resembling bits of cheese were passed, and occasionally shreds or membraniform substances escaped during the act of defecation. In many instances neither fœces nor blood were evacuated, the dejections consisting merely of a white "slime or jelly," and entirely inodorous. When scybalæ were passed, the patient experienced temporary relief from the tormina and tenesmus and other distressing symptoms. The violent and ineffectual efforts to evacuate the bowels would occasionally, especially in cases of children, cause prolapsus ani, or protrusion of the coats of the gut beyond the verge of the anus, which would add greatly to the distress of the patient. I remarked, in nearly every instance, that the desire to evacuate the bowels was periodical, or, more properly speaking, paroxysmal; that one evacuation would beget the desire to have another, for a succession of five or six times, when the patient would lie quiet and comparatively easy for the space of one or more hours, when an irresistible inclination to go to stool would be experienced; and when placed upon it he would continue to strain for the space of a quarter or half an hour, or until he was forced by persuasions to assume a horizontal posture; nothing during the whole time being voided except a little bloody mucus and flatus. Usually there was great heat in the abdomen and about the head. The thirst was intense and almost unquenchable. Except during the period of remission the pulse was seldom below 100, generally ranging from 110 to 130 per minute. The tongue was generally loaded with a dark crust or tenacious fur, and sometimes fissured; and when the crust peeled off it left the tongue of a fiery red, smooth, and glossy appearance. The inner surfaces of the lips, dorsum of the tongue, and fauces were mostly dry and cracked, and the countenance, as in most abdominal affections, indicated excessive oppression of the nervous system, with general debility, and great oppression of the vital forces and energies. The fever which accompanied the disease was mostly remittent in its type, though it often assumed a synochal or continued form, merging, if the disease ran a persistent or protracted course, into a low grade of febrile phenomena, called, generally, *typhoid.* In a few cases typhoid symptoms supervened in the first instance, and accompanied the disease throughout, rendering its course very tedious and the convalesence protracted. I saw but few urgent cerebral symptoms among the numerous cases which came under my care. During the exacerbation of the fever, patients would occasionally exhibit delirium, manifested by low muttering and incoherent talking, especially when asleep; but as soon as they were aroused from their temporary slumbers they would appear rational. There was no coma nor stupor present; on the contrary, patients were sleepless and watchful, and very apprehensive and fearful of a fatal termination. The skin, except about the head and over the abdominal and thoracic viscera, was uniformly cool—not above, if equal to the ordinary temperature of the animal body. Perspiration was common, but unaccompanied with any mitigation of the complaint or any amelioration of the febrile symptoms; it did not appear to be a critical evacuation, nor was it accompanied or followed by a cessation of disquietude and jactitation; but it had no tendency to abate thirst or allay the restlessness of the patient. I have seen patients bedewed with copious perspiration while the mouth, tongue, and fauces were perfectly dry, and the teeth covered with thick sordes. With the exception of two cases which I treated, one an old and the other a middle-aged negro man, the pulse was uniformly quick, irritable, and weak. In these two cases the pulse was inordinately slow, indeed much below the usual standard of health, and yet in both the disease was not amenable to remedies, and ran a long, persistent, though favorable course; although at no stage of their illness did either of these cases present any threatening symptoms or evince much malignity.

I have thus given, in a hurried and disjointed manner, a brief outline of some of the prominent symptoms which marked the prevalence of the epidemic, but I feel that my task would be unfinished without a detail of the symptoms present in some of the fatal cases which occurred in the family of one of my former patrons, John Thompson, Esq., a worthy and gentlemanly planter, residing some six or seven miles east of this place. The symptoms in these cases differed materially from those above described, and resembled in many respects those of malignant cholera. Indeed, I think if they had occurred in a cholera district they would have been so regarded by the most competent medical judges, especially if they had been disconnected with cases of epidemic dysentery. In this family, where the subjects of the epidemic were confined almost exclusively to little negroes, and all of whom had previously been attacked with scarlatina, and labored under its usual sequelæ, such as dropsical effusion into the cellular tissue, etc., and by measles, the disease evinced a stubbornness, a malignity, and a fatality which I never before witnessed. The materies morbi, or infectious effluvia, emanating from the bodies of these patients became so virulent and concentrated as to generate disease approximating, as just stated, in its general symptoms and pathological appearances, closely to cholera. These patients were crowded together in a small apartment, and four of them died. Three of the fatal cases occurred in the same family—two brothers and a sister. The attack in each of these was ushered in by nausea and vomiting, and profuse rice-water evacuations from the bowels, intense thirst, great prostration of the vital energies, coolness of the trunk, and coldness of the extremities. The centrifugal force seemed to be entirely counteracted by the centripetal; the entire periphery or superficies of the body being cool. The pulse was very irritable, frequent, and so feeble as to render it almost imperceptible at the wrist. The features were pinched; the eyes sunken; the cheeks hollow; the mouth and fauces dry; the tongue cold. These patients were extremely restless, complaining constantly of great internal heat, throwing off the covering from their bodies, and crying incessantly for cold water. Nothing could restrain the profuse rice-water evacuations, which they continued to discharge until they died. Death was preceded in three of these cases by genuine cramps of the abdominal muscles. At the time these cases occurred there were in the same family several cases of unmistakable dysentery, which ran through the usual stages of the disease, and terminated favorably. With the exception of these four cases, the phenomena of which were veiled in obscurity, the writer saw no cases materially deviating from ordinary epidemic dysentery. I understand a number of these anomalous attacks took place in the practice of other physicians in the surrounding country, all of which, without a solitary exception, proved fatal. Dr. Bowen, of Lawrenceville, Alabama, some eighteen miles northwest from Fort Gaines, in whose neighborhood the visitations of epidemic dysentery were truly terrific, encountered, I understand, several of these abnormal cases not at all dissimilar to, but in every respect resembling those which occurred in the family of Mr. Thompson.

*Pathology of Dysentery.*—Dysentery is essentially an inflammatory disease. The difference between *sporadic* and *epidemic* dysentery is that, in the former the fever is merely symptomatic of inflammation of some portion of the intestinal tube, while in the latter a constitutional disease is engrafted upon the local one, being marked by symptoms of great prostration and general debility, rendering it a most fearful complication, and giving rise to the malignity of symptoms which characterize its progress. The disease under such circumstances becomes highly infectious, making it communicable from one person to another, especially when congregated together in small and filthy apartments.

Epidemic dysentery does not exist as a local affection *per se*, but to this local affection is superadded or conjoined a general disturbance of all the vital actions and functions of the body. The inflammation has its location or seat in the colon, hence most of our modern medical authors describe it under the name of colitis. Pathological anatomy has revealed this as the seat of the complaint, but the inflammation is not, however, invariably confined to the colon. The mucous coat of the latter may at first be the focus, but the inflammation radiates or spreads to contiguous parts of the intestines until considerable portions of it are involved in the general phlogosis. An opportunity was afforded me but once, during the prevalence of the late epidemic, to inspect the morbid appearances exhibited after death: and the patient whom I assisted to dissect and examine was one of the four who died with the anomalous symptoms heretofore described as approximating to those of malignant cholera. In this case the anatomical characters of cholera were strongly marked. I need not enumerate them, having already consumed too much time in the symptomatology of dysentery.

The general cause of the late epidemic was, in all probability, a peculiar constitution of the atmosphere, in some instances following, in others accompanied by the measles,—the latter generally, as is well known, a mild and not very dangerous disease when appropriately treated and uncomplicated with other affections, but when associated with dysentery often one of the most intractable and unmanageable complaints imaginable. The *exciting* causes of epidemic dysentery may be sudden changes of weather, cold and moisture, acid and unripe fruit, drastic cathartics, etc.; but, in order to give these potency of action, there must be some such predisposition as exists in persons whose vital powers have been previously depressed by fatigue, watching, anxiety, breathing an impure or infectious air, feeding upon an unnutritious or unwholesome diet, drinking bad water, nocturnal exposure, etc. In most of the cases which we witnessed here, during the prevalence of the epidemic, the cause could be distinctly traced to a peculiar state of the alimentary canal, left by a previous attack of measles. If it did not make its appearance immediately after the subsidence of measles, yet those persons who had suffered from an attack of the latter, even though some weeks had elapsed from the time of their recovery to the invasion of dysentery, were peculiarly liable to it, and endured it with less tolerance than those who contracted it without having been the subjects of the former.

As a matter *pro formâ*, I suppose I ought to say something in regard to the diagnosis and prognosis of dysentery; but, as I shall extend this paper to a much greater length than I originally intended, I must necessarily be brief on these two points.

*Diagnosis.*—It is almost impossible to confound epidemic dysentery with any other complaint. Its peculiar features and characteristics are so well marked and determined, that it is not likely to be mistaken for anything else, or *vice versâ*. Its true nature is known not only by the profession, but even the ignorant and uninformed portion of our population recognize it. The only malady which may possibly be mistaken for dysentery is hemorrhoids, on account of the tenesmus and the bloody mucous discharge; but the pain and soreness around the verge of the anus, and, above all, the presence of the hemorrhoidal tumors themselves, to say nothing of the absence of the constitutional symptoms, such as fever, etc., will remove all doubt whatever.

*Prognosis.*—This may be regarded as favorable when there is a general subsidence of all the severe and urgent symptoms; when the fever abates, and the pulse moderates in frequency; when the skin becomes equably warm and moist; when the inside of the mouth and fauces, and the tongue, cease to be dry; when the intensity of thirst diminishes; when the heat, soreness, and tenderness in the abdominal region begin to give way; when the tongue assumes a healthy appearance; when the appetite of the patient begins to return; and, above all, when the stools, after having been bloody and mucous, become bilious-feculent, and the tenesmus and tormina abate. On the contrary, if the abdomen becomes tumid or tympanitic; the skin moistened with a cold, colliquative perspiration; the eyes sunken in their sockets; the cheeks collapsed; the jaws fallen; prostration extreme; ejections involuntary; pulse rapid, quick, thready, weak, and almost imperceptible; low muttering delirium; incoherent speech; great confusion of the intellectual faculties, and subsultus tendinum, the prognosis is unfavorable.

Before I proceed to a short history of the course of the epidemic, it may not be inappropriate or out of place to furnish the readers of this article with a short topographical and historical sketch of Fort Gaines, the county seat of Clay County, Georgia. To the medical philosopher, and especially to the ætiologist, topographical descriptions of certain districts of country, and even of particular localities, form an inviting field of study. Materially differing but little from, I shall regard Fort Gaines as the representative type of the surrounding country. The Chattahoochee river taking its rise in the northern or mountainous parts of Georgia, and, running from north to south, until it disembogues itself into the Gulf, through Apalachicola Bay, forms the dividing line between this and the State of Alabama. At Fort Gaines the river forms a curve, at the angle of which rises a high pine bluff, composed of marl and other cretaceous substances. This bluff is one hundred and seventy-eight feet above low-water mark, and nearly perpendicular, and on its summit, opposite the convexity of the curve of the river, lies the village of Fort Gaines, containing a population of only four or five hundred inhabitants. The village is situated a little north of 31½° N. latitude, on the eastern bank of the river. Standing on the bluff, at the immense height of one hundred and seventy-eight feet above the water, and commanding a view of the river as it winds its course slowly along the base of the bluff, with a beautiful landscape intervening between the west bank of the stream and the adjoining hills in the distance, it presents to the spectator a most picturesque and romantic appearance. Spreading out in an easterly direction from the bluff is a level sandy plateau, half a mile in width, bounded by pine hills rising, according to the calculations of our former State Geologist, Dr. Cotting, to the height of one hundred and forty-eight feet above the level on which the village is located. The town still goes by the name by which it was called when a military post. The barracks were erected here in 1817, for the protection of the United States troops against the attacks of the hostile Indians, and originally stood on the bluff near the river. In the concavity of the curve on the opposite side of the river a swamp or lowland, about a mile in width, extends to the high hills on the west. This bottom was originally wet and marshy, and filled with lagoons, but is now in a high state of cultivation. On the Fort Gaines side there is no swamp, with the exception of a narrow belt of bottom land, lying above the village, and running parallel with the meanderings of the river. The bluff on the eastern side of the river is elevated perhaps more than one hundred feet above the west bank, which gives the spectator a commanding view, as he stands upon the summit, and overlooks the lowlands lying between the river and the high western hills by which they are limited. The face of the country, like most "long leaf pine" tracts, is level, except on the sides of brooks, creeks, etc., where it is not only undulating, but, in many places, steep and precipitous. Geologically considered, the soil near the surface is sandy, with clay intermixed, covering, in some places, a marl foundation abounding in cretaceous formations and other heterogenous products. At Fort Gaines there is no clay in the soil, hence it is not well adapted to cultivation. Manures are of no permanent benefit to the soil, which, on account of its porous, sandy character, suffers all fertilizing fluids to percolate it, producing, unless frequently reapplied, but little good effect. There is a substratum of impure carbonate of lime underlying the surface, at indefinite depths, which is not by any means uniform in its distribution Water passing through these calcareous beds becomes strongly impregnated with lime, and has, to those unaccustomed to its use, a very disagreeable taste. The original and characteristic forest tree of the plain on which the village stands, and which prevailed to the exclusion of almost every other wild growth, was the long-leafed pine. But few of these "natives" of the forest now exist, having long since been felled, and their places supplied by ornamental trees. A species of evergreen oak, closely allied in its habits to the live-oak, and called by us, in common parlance, *water oak*, has been transplanted to the yards and sidewalks, and affords in the heat of summer a cool and dense shade. But few sources of malaria exist; yet the prevailing epidemic diseases are those which are supposed to be produced by that subtle and inappreciable agent, such as intermittent and remittent fevers. The type of one or other of these fevers impresses itself upon nearly all of our inflammatory complaints. Hence, in pneumonia, pleurisy, dysentery, and all that class of ailments where the phlogistic diathesis is usually most fully developed, instead of synochal we generally have remittent, and sometimes intermittent fever, as an accompaniment. Our climate is variable, the transitions from heat to cold, and *vice versâ*, being sometimes exceedingly sudden. Our summers are very long, with a temperature generally ranging from 78° to 94° Fahr. in the shade. Our winters are temperate, mild, and pleasant, with the exception of occasional "cold spells," when north winds prevail. Their common temperature ranges from 48° to 62°. I must now return to a history of our late epidemic, and then close this paper with an account of the treatment employed.

*History of the Epidemic.*—The year 1857, up to this date, (November 2,) has been, in this section, at least, remarkable for a peculiar epidemic constitution of the atmosphere, which has prevailed, to a greater or less extent, since the month of January. During this time we have had four epidemic visitations in the following order of succession:—1st. Epidemic Scarlatina: limited, however, comparatively speaking, to a few neighborhoods. 2d. Epidemic Measles, which prevailed more or less extensively over a large extent of country. 3d. Epidemic Dysentery, which was general in its prevalence, and more or less fatal in its ravages. 4th. Epidemic Catarrhal Fever, which is now the prevailing disease. The first-mentioned, scarlatina, made its appearance in the month of January, and continued, with more or less violence, until the summer months. In the month of February, rubeola appeared, and continued through that month, March, April, May, and a portion of June. But little fatality accompanied these two epidemics, except in cases complicated with other diseases, or with local inflammation of vital organs. The most usual complications were bronchitis, pneumonia, dysentery, and diarrhœa. Whenever any of these inflammations supervened upon or accompanied the measles, the disease was formidable to manage. The meteorological changes of the weather have been the most extraordinary and fitful ever experienced. On the 18th and 19th of January, we experienced an intensity of cold never before felt in this latitude—the mercury descending to 6°, in the open air, and to 10° within doors. During that intensely cold spell I had sixteen scarlatina patients under treatment in one family.

The month of February was unusually warm for the season; the weather was so temperate that most of our old farmers predicted an uncommonly forward spring, and went rapidly to work, making preparations for planting; but the month of March was ushered in with cold winds and frosty weather, and continued cold throughout. The month of April was likewise cool, and even cold on some days. About the first of May the barometrical changes were very great, as well as the thermometrical. On the sixth day of that month we had a most terrific hailstorm, which literally ruined the crops throughout its entire range. On the 15th, another hailstorm devastated portions of the country. A great many hailstorms—indeed an unprecedentedly great number for the length of time—swept over the country, doing an incalculable amount of mischief to crops and other vegetation. Most of these hailstorms were accompanied with strong gusts of wind and rain, and pursued a course invariably from the northwest to the southeast. Our summer was a very short one. From May until the 20th of July we had a protracted drought. The rainy season then commenced, and continued until nearly the 1st of September, during which time we had showers nearly every day, and occasionally some heavy falls of rain. Early in the month of June the epidemic dysentery appeared, first in persons who had recently recovered from attacks of measles. In some places, the measles and dysentery prevailed together, in which case the dysentery was almost uncontrollable by remedies, and malignant in the extreme, decimating entire neighborhoods, and almost destroying whole families. In the neighborhood of Lawrenceville, Alabama, it was characterized by a most fearful and alarming fatality; scores upon scores of persons, including all ages, sexes, and colors, fell victims to its ruthless attacks. It has now nearly ceased as an epidemic, only a few scattering cases being occasionally met with. After a careful analysis of all the circumstances connected with this epidemic, I think the natural inference deducible from the premises is in favor of a peculiar condition of the atmosphere, as the cause of the visitation—a condition indicated by the unusual barometrical and thermometrical changes already alluded to, as well as by the presence of some subtle agent whose influence was widely exerted and disseminated throughout extensive tracts of country. What that peculiar poisonous agent was, I am unprepared to say; but there was evidently a connection between it and the meteorological causes which gave it such a wide diffusion.

*Treatment.*—The treatment of the disease, which, from experience, I found most successful, was the following—viz.: If called to the patient soon after the onset of the attack, a few grains of mercury with chalk and Dover's powder, were administered—say five grains of each, repeated at intervals of two or three hours, until two or three portions had been exhibited. If the stomach was very irritable, and would not tolerate the Dover's powder, the sulph. morphia, in proportional quantities, was substituted for it. At the expiration of a few hours this was followed with some of the neutral salts, most generally sulph. magnesia, dissolved in an infusion of capsicum or ginger. After two or three operations from the bowels had been procured by means of the salts, the following combination was administered, to adults in tablespoonful doses, and to children in teaspoonful doses, repeated every hour or two, according to the urgency of the symptoms and the frequency of the evacuations:—

℞.—Tinct. rad. rhataniæ,

" camphoris, āā ʒij;

" capsici,

" opii, āā ℥j; craetæ preparatæ, gum acaciæ, sacch. albi, āā ℥ss; aquæ, ℥viii.

A combination of castor oil and turpentine was a favorite remedy with some of our physicians, as a cathartic, in the initial stage of the disease; but from the experiments I made with it, I soon became satisfied that it was not superior, nor even equal to the epsom salts; its effects were too drastic.

Soon after the late Dr. James Johnson, of London, published his works on the *Diseases of the Liver and Tropical Climates*, our southern physicians ran wild on the subject of calomel. These works were regarded as the highest medical authority in the South, and the library of no southern physician was considered complete without a copy of them. They were consulted as oracles in theory and practice, and from the doctrines there inculcated calomel came to be considered as a *sine qua non* in the treatment of nearly all our southern febrile diseases, and was prescribed with but little discrimination. Hence a great many southern physicians still adhere to it, and the practice of treating epidemic dysentery with a combination of calomel and opium has been too generally adopted. But I trust the day is not far distant when calomel will be no longer employed in the treatment of this disease. In my judgment, it is not adapted to the cure of dysentery, and this opinion is predicated upon careful observation of its effects at the bedside of the sick. I do not mean to be understood as condemning the use of this drug in sporadic dysentery; on the contrary, I think under certain circumstances it is a valuable remedy, and one from which the best effects have frequently resulted; but I do most solemnly protest against its use in epidemic dysentery. An incalculable amount of mischief, I fear, has resulted from its injudicious and indiscriminate employment in the treatment of this disease. If admissible at all, it is only so in alterative doses—viz. in minute quantities. When otherwise given in this affection, I have often seen it produce nausea, vomiting, griping pains, and great faintness—symptoms which rapidly subside upon a discontinuance of the remedy. The only preparation of mercury which I think can be safely given in epidemic dysentery is the hydrargyrum cum creta, and I regard this as almost indispensable in the management of this affection. I have seen bad effects result even from the employment of blue mass, a fact which has created in my mind a distrust of the propriety of administering it in epidemic dysentery.

I have said this much in condemnation of the "calomel practice" for the purpose of warning my professional brethren against a too great reliance on its powers as a therapeutical agent in the treatment of epidemic dysentery; for I am persuaded that the remedy is too great an irritant of the mucous coat of the intestines.

When the stomach will tolerate the use of Dover's powder, I have seen the best effect accrue from a few grains of hydrargyrum cum creta, in combination with the former, repeated two or three times per diem, especially if the patient can be induced to take freely of warm diluent drinks. The secretions from the liver are by this means promoted; the pain and irritation of the intestinal canal abated; the functions of the perspiratory exhalants increased; and the restlessness and general disquietude relieved. Opium, or some of its preparations, I consider the best of all remedies in epidemic dysentery. It is the "Magnum Dei Donum," for without it we could not successfully control the pain, griping, frequent alvine evacuations, and general disquietude under which patients suffer. It has a most soothing and tranquillizing effect upon the nervous system, and when conjoined with the acetate of lead in doses of one-half to a grain of the former with $2\frac{1}{2}$ grains of the latter, repeated at intervals of two or three hours, according to the urgency of the symptoms, it forms a most reliable dependence. It lessens the peristaltic action of the intestines, thus checking the discharges, and thereby giving rise to the impression, which prevails among some of our physicians at the South, that it is *de facto* a curative agent instead of being an adjuvant to other remedies.

As an external application in the treatment of dysentery, warm mush poultices, made stimulating by mixing mustard or finely powdered cayenne pepper in the mush, and applied as warm as the patient can bear them, form a most soothing and comfortable application. To procure gentle alvine evacuations from the bowels, which I think is highly necessary in the treatment, I know of no medicine of equal value with the sulphur and cream of tartar. It should be administered in cathartic doses, about every three or four days, and repeated until feculent discharges are procured. Many of our physicians prefer salines, such as the neutral salts; but I give sulphur and cream of tartar a decided preference.

Throughout the treatment of the late epidemic I also found quinine a most valuable remedy. Indeed, without its use we could not have controlled the accompanying fever. The latter was most commonly of the remittent type, the periods of abatement and exacerbation occurring every 24 hours. The abatement commonly took place early in the morning, and the exacerbation about 10 o'clock in the forenoon, the acme being usually from 2 to 4 o'clock in the afternoon, and the stage of declension from 9 o'clock at night until daylight the next morning. To cut short the paroxysm, which could not be entirely prevented by the administration of the quinine, or, at least, to mitigate the severity of the febrile symptoms, I most usually commenced the use of the remedy about 9 o'clock at night, and exhibited it until 9 o'clock the next morning, in doses ranging from 3 to 5 grains, repeated every two or three hours.

In most cases the functions of the stomach were entirely suspended, so that substances taken in the solid form would sometimes lie in that organ for several hours without undergoing scarcely any chemical change whatever. Under these circumstances, and with a knowledge of this fact, I seldom exhibited quinine in the pilular form. The formula which I generally used was the following :—

℞.—Quiniæ sulph. ℨi ; acid. sulph. gtt. xxx ; aquæ, ℥vi.

Of this solution I generally gave a large tablespoonful every two or three hours.

Dr. McHath, of Arkansas, in the October No. of the *Southern Medical and Surgical Journal*, invites the profession to a trial of creasote. In one or two hundred cases treated by him and his partner during the present year, he found that it was *the* remedy. He speaks of it in terms of unbounded commendation, and extols it as being almost a specific in epidemic dysentery. His formula is the following :—

℞.—Creasote, gtt. x ;
acid. acetic. gtt. xx ;
morphiæ sulph. grs. ii ;
aquæ, ℥i.

Of this a teaspoonful was administered every two or three hours. I regret very much that I had no opportunity, during the prevalence of the epidemic here, of testing the power of this article. Dr. Fowler, of this place, informs me, however, that he treated several cases of the disease with the creasote, and that in every case it acted "like a charm." Dr. Paullin also speaks favorably of it as a remedial agent. From the testimony of these three gentlemen, and others, who gave it a fair trial, I am disposed to think highly of it, and as such bespeak for it a fair, honest, and impartial trial by the profession.

In the treatment of the epidemic under consideration, our physicians encountered immense difficulties in consequence of the meddlesome interference of officious friends and acquaintances. There was scarcely a man or even an old woman in the community who did not possess some specific, some sovereign remedy for the cure of the disease, and who, in the kindness of their hearts, and with generous impulses seldom witnessed, would not only urge upon patients or their friends the necessity of "quitting the doctors," but of trying their simples. To enumerate all their remedies would be out of place here ; but the following may be mentioned as the principal—viz. white sumac, chinquepin-root tea, white-oak runner tea, sweet-gum bark tea, pine bark tea, red shank tea, post-oak bark tea.

A combination of camphor, paregoric, and number 6 was also much in vogue among the common people, and the following prescription was often vauntingly used, to the displacement of almost every other means:—

℞.—Camphor, opium, cayenne pepper, āā ℨi; brandy, oij.

Dose a tablespoonful every 2 or 3 hours.

This preparation was used indiscriminately throughout every stage of the disease, and was the only one of their remedies which possessed any merit whatever.

Besides the means above mentioned, enemata of flaxseed tea, laudanum and starch, nitrate of silver, tincture of iodine, cold water, or even ice-water were found valuable adjuvants in the treatment of the epidemic. I found enemata of ipecac mixed with flaxseed tea or starch, to which laudanum was added, answered a better purpose. I have seen the happiest effects follow the use of this remedy, when thus administered, and can confidently recommend it to the profession as of the greatest value. It promptly relieves the tormina and tenesmus, allays irritation of the mucous coat of the intestines, and checks, sometimes for several consecutive hours, the evacuations from the bowels. When the fever was intense, accompanied by urgent thirst, great restlessness, and inordinate heat of the abdomen, enemata of cold water, and even ice-water, were followed by the most soothing and happy effects. In these cases I often substituted, for the warm mush-poultices, large towels dipped in ice-water and frequently applied over the abdominal region, often using at the same time injections of ice-water.

Throughout the treatment, opium, Dover's powder, or morphia entered into almost all of our prescriptions. The hydrargyrum cum creta was used when a mercurial seemed to be indicated. When the disease ran, as it often did, a protracted course, and the powers of the system began to flag, brandy, turpentine, ether, and other diffusible stimulants were found to be absolutely necessary. Dr. Stokes's assertion that in all inflammatory diseases there is a stage when stimulants act as antiphlogistics, is peculiarly true of inflammation of the intestines. Hence brandy is not only admissible, but indisspensably requisite, and was successfully used by us in quantities dependent upon the effects it produced. A number of our physicians placed great reliance upon the application of blisters. I cannot say that as a general rule I entertain a very exalted opinion of their efficacy. When all other means prove inadequate and unsuccessful, and their application is delayed until the "blistering stage" of the disease arrives, I have no doubt but that they often do good; but unfortunately they are frequently employed too early, and their application is often attended with injurious if not fatal effects. When resorted to they should not be allowed to remain on until full vesication is produced, as they often give rise to the most painful strangury. When they produce a tolerably powerful rubefacient impression they should be removed and a warm poultice applied over the surface.

